# Developing a Conceptual Framework: Integrating Tumour, Macroenvironment and Resource (TMR) as a Composite Determinant of Ovarian Cancer Outcome in Diverse Resource Settings

**DOI:** 10.3390/cancers18203299

**Published:** 2026-10-13

**Authors:** Asima Mukhopadhyay, Adam Ruddock, Mohamed Elsebaei, Ally Memon, Daity Bhattacharjee, on behalf of the Tumour-Macroenvironment-Resource (TMR) Global Review Group

**Affiliations:** 1 Teesside Ovarian Oncology Research Group (TORCH), Department of Gynaecological Oncology, University Hospital Tees, Middlesbrough TS4 3BW, UK; adam.ruddock@nhs.net or a.l.ruddock@northumbria.ac.uk (A.R.); mohamed.elsebaei@nhs.net (M.E.); 2 Kolkata Gynecological Oncology Trials and Translational Research Group (KolGOTRG), Kolkata 700156, India; 3 Faculty of Society and Culture, Northumbria University, Newcastle upon Tyne NE1 8ST, UK; ally.memon@northumbria.ac.uk

**Keywords:** ovarian cancer, macroenvironment, resource

## Abstract

**Background:** Ovarian cancer (OC) poses an escalating global health burden, with marked disparities in incidence, mortality, and survivorship. Social, psychological, and structural determinants, including deprivation, stigma, and access to specialist care, may influence outcomes but are not well-represented in existing models of cancer care. We introduce the Tumour, Macroenvironment and Resource (TMR) framework, a novel conceptual model exploring interactions among tumour biology, social determinants, and healthcare resources in OC survivorship and care. **Methods:** Framework development occurred in three phases. First, a narrative review identified factors influencing OC outcomes across tumour, macroenvironmental, and healthcare resource domains. Second, multi-criteria decision analysis (MCDA) was used to structure these factors within a preliminary framework and assign indicative domain weightings. Third, two cross-sectional expert opinion surveys were conducted to refine and validate the concept of the framework. The final model was then evaluated in a retrospective pilot cohort to assess the feasibility of collecting predefined TMR variables from routinely available clinical data. **Results:** The review identified limited evidence addressing broader determinants of health beyond biological prognostic factors. Available studies suggested interactions between tumour characteristics, social determinants, and healthcare infrastructure that may influence outcomes. A preliminary model allocated 50% weighting to tumour factors and 50% to combined macroenvironmental and healthcare resource factors. Expert surveys (*n* = 20 and *n* = 44) achieved consensus and informed development of the final TMR framework. Application to a UK pilot cohort of patients with OC *(n* = 42) demonstrated the feasibility of collecting variables across all TMR domains and identified multiple adverse factors across tumour, macroenvironmental, and resource domains. **Conclusions:** The TMR framework provides an integrative, hypothesis-generating model for exploring disparities in OC survivorship and care. Further retrospective and prospective validation are required before clinical or prognostic applications can be considered.

## Simple Summary

This paper presents a conceptual framework for understanding ovarian cancer survivorship, challenging the traditional view that tumour-specific factors alone shape patient experiences and outcomes. While tumour biology, stage and treatment responses are critical considerations, we propose that survivorship is also influenced by broader external factors. These are organised into the following two domains: macroenvironmental factors and resource-based factors. Macroenvironmental factors include social determinants of health such as socioeconomic status (SES), social support and culture. Resource factors refer to healthcare setting characteristics, including the availability of a skilled workforce, infrastructure and treatment technologies, which vary across different resource settings. The proposed Tumour, Macroenvironment and Resource (TMR) framework provides a conceptual structure for considering how these domains may interact in ovarian cancer, which has the highest mortality rate among gynaecological cancers globally. This framework highlights areas for future research and may support efforts to better understand disparities observed across healthcare systems.

## 1. Introduction

Gynaecological cancers represent a significant global health crisis, imposing substantial burdens on healthcare systems, organisations, and patient populations worldwide [1]. Global trend analyses demonstrate alarming increases in incidence and mortality. For example, the incidence of cervical cancer rose by 62.9% between 1990 and 2021, with associated deaths increasing by 40.2% [2]. Similarly, ovarian cancer incidence more than doubled between 1990 and 2019, while the overall female cancer age-standardised incidence rate (ASIR) increased from 47.1 to 50.7 per 100,000 population [3]. According to the GLOBOCAN 2022 forecast, the annual number of women diagnosed with OC is forecast to increase by over 55% by 2050 [4].

Clinical outcomes in oncology are influenced by a complex interplay of socioeconomic, demographic, and health system factors across diverse care settings. Structural inequities, including political context and geo-political conflicts, healthcare investment, and the sociocultural status of women, profoundly shape gynaecological oncology outcomes globally. Cultural and religious stigmas surrounding gynaecological cancers, often linked to sexuality or sexually transmitted infections, further exacerbate underreporting and late presentation of disease [5].

OC, which has the highest mortality rate among gynaecological malignancies [6], remains particularly challenging due to late-stage presentation and diagnosis and unequal access to specialist cytoreductive and targeted therapies [7]. Critical yet under-addressed determinants include social and economic deprivation, demographic disparities, and psychological dimensions such as hope, fear of recurrence, will to survive, and quality of life (QoL) [8].

Although advances in early detection, diagnosis, and innovative treatments have improved outcomes for many OC patients, survival rates remain unacceptably low, particularly within diverse resource-stratified settings. Whilst this disparity exists across a global context, local disparity exists in pockets within high-income countries particularly amongst women from low socioeconomic backgrounds and cultural minorities facing barriers related to healthcare access, language, and education [7,9].

The consequences of this global health crisis extend beyond physical morbidity to encompass emotional distress and inequitable access to effective interventions, including prehabilitation and rehabilitation services [10]. Persistent disparities in timely diagnosis, access to care, adherence to treatment, and psychosocial wellbeing highlight the multifaceted nature of inequality across the cancer care continuum [11].

This paper introduces the novel concept of TMR (Tumour Macroenvironment Resource), a conceptual framework designed to explore how tumour-related, macroenvironmental and resource-related factors may interact to influence cancer survivorship and care in gynaecological oncology. The concept was initially informed by clinical experiences, observations, case series and root-cause analyses conducted in resource-adapted settings, where variation in patient experiences and outcomes was observed despite apparently favourable biological characteristics. These observations served as the basis for development of a hypothesis-generating framework requiring further formal evaluation and validation. The concept of macroenvironment and resource factors in addition to anatomical tumour in cervical cancer has been mentioned in the consensus statements from the GCIG [12]. To our knowledge, this is the first structured presentation of the TMR framework in ovarian cancer, incorporating a tabulated conceptual model and proposed weighting structure derived from a narrative review and refined through expert consensus. The TMR framework is not currently a prognostic score but a conceptual framework with a prototype operational scoring architecture.

The present study aims to establish its theoretical basis, evaluate expert acceptability, and assess the feasibility of measuring its constituent domains using routinely available clinical data. The proposed weighting and scoring structure should therefore be considered exploratory. Subsequent studies will be required to determine construct validity, empirically appropriate weighting, prognostic performance, and clinical utility.

## 2. Materials and Methods

**Figure 1:**
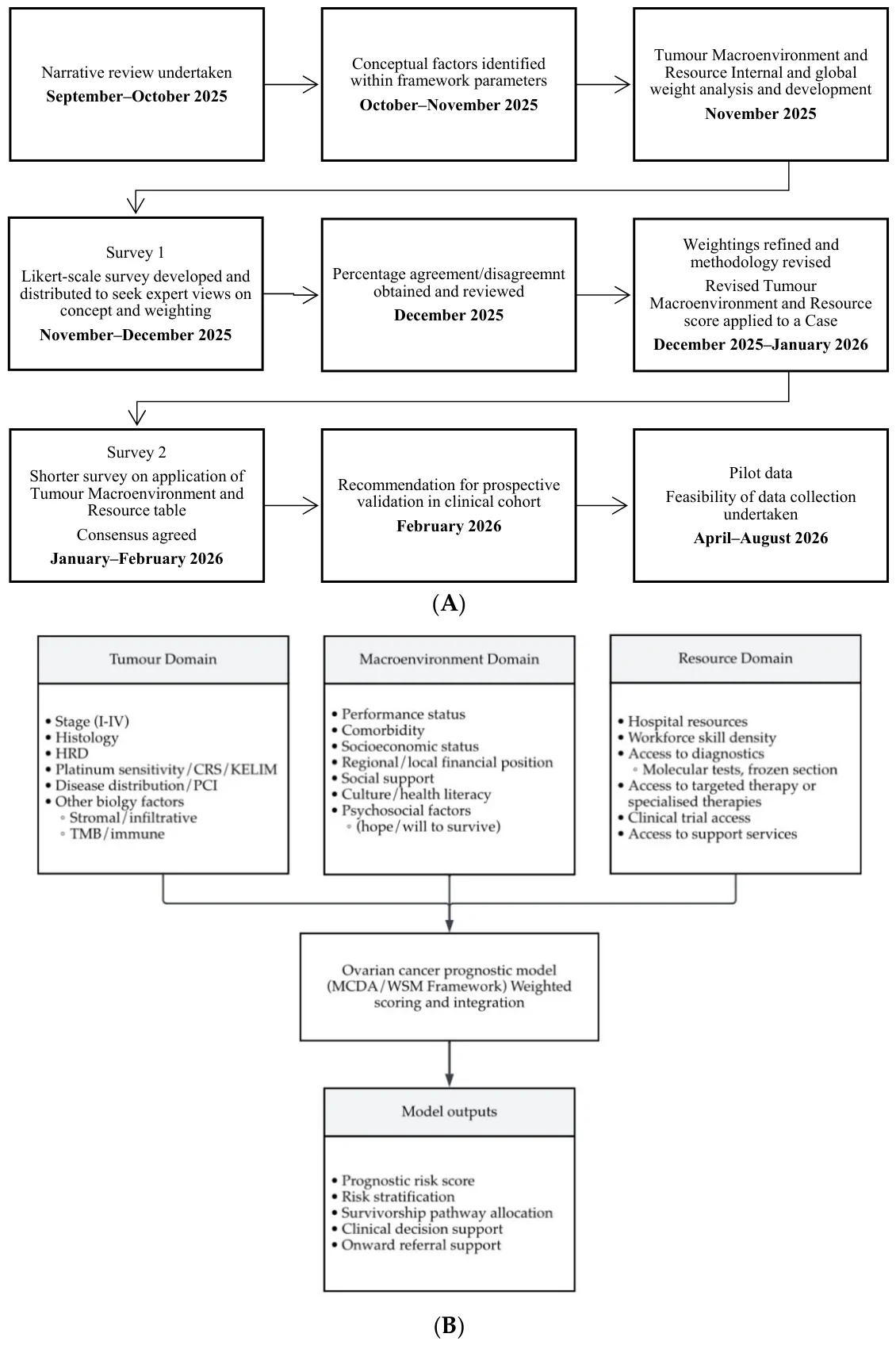
Pictoral representation of the conceptual development process. (**A**) Flowchart of the development and refinement of the Tumour Macroenvironment and Resource framework with timescales, including narrative review, expert survey consensus, weighting analysis, methodological revision, case application, and recommendations for prospective clinical validation. Figure created by the authors. (**B**) is a conceptual chart/graphical abstract demonstrating the Tumour Macroenvironment and Resource framework and the factors within each domain in a visual format including model outputs. Figure created by the authors.

This study employed a multi-phase mixed-methods design comprising: (1) a narrative literature review and conceptual synthesis; (2) development of the Tumour-Macroenvironment-Resource (TMR) framework; (3) expert review and consensus through two cross-sectional opinion surveys; and (4) feasibility assessment of the framework using a retrospective pilot cohort. The objective was to develop and achieve expert consensus on a conceptual framework integrating tumour, macroenvironmental, and healthcare resource factors relevant to ovarian cancer outcomes. Figure 1A,B illustrate the structured conceptual synthesis of the Tumour-Macroenvironment-Resource (TMR) framework.

### 2.1. Narrative Review and Conceptual Synthesis

Narrative and scoping reviews are designed to map the breadth and scope of research across diverse study types and disciplines [13] with a view to synthesising evidence and identifying critical knowledge gaps. A narrative review and conceptual synthesis approach was adopted to explore the relationship among tumour biology, macroenvironmental factors, resource settings and survivorship in gynaecological oncology through the lens of the proposed TMR conceptual framework for understanding survivorship and care in gynaecological oncology. The relevant literature was identified through a comprehensive search of peer-reviewed databases including PubMed, Scopus, Science Direct and review of Lancet oncology publications in gynaecological oncology and women’s health. Search terms included “gynaecological oncology”, “ovarian cancer”, “social determinants of health”, “health disparities”, “survivorship” “high/low resource settings” and “macroenvironmental factors”. The grey literature, including government and policy reports, were searched via Google Scholar and organisational databases such as World Health Organisation (WHO) to offer a complete picture of the approach to cancer survivorship models across diverse tumour groups and provide the relevant literature linking TMR factors to cancer policy. Within the narrative review, existing clinical and conceptual frameworks were reviewed with an emphasis on contextual and systemic domains to explore how biological, social and healthcare factors have been considered across diverse tumour groups. These existing frameworks combine diverse data types and seek to improve understanding of factors associated with survivorship, however; they fail to incorporate MCDA beyond traditional biological factors. A Scale for the Quality Assessment of Narrative Review Articles (SANRA) checklist was completed (Supplementary Document S1).

### 2.2. Narrative Review Criteria

Inclusion criteria comprised studies published globally that examine social, structural, or clinical factors influencing outcomes in gynaecological cancers with a particular focus on ovarian cancer. The review examined social, environmental and systemic determinants influencing clinical or psychosocial outcomes. The information presented empirical findings or conceptual analyses relevant to survivorship, access to care and quality of life. Exclusion criteria included editorials, conference abstracts and studies lacking empirical or theoretical grounding. Additionally, existing evidence of traditional biological diagnosis of cancer and staging was accepted and excluded.

To develop the conceptual TMR framework, data were extracted using a structured template capturing study type and setting and imported into EndNote for citation management and duplicate removal. Extracted data were synthesised using thematic analysis informed by the novel conceptual framework of TMR. This framework facilitated organisation of findings into the following three overarching domains: (1) clinical determinants (2) social and structural determinants, and (3) resource and setting factors. Patterns and relationships were mapped to highlight evidence gaps, cross-setting disparities, and the interplay among resource availability, survivorship and cancer care experiences.

Given the extensive research base on tumour characteristics in gynaecological cancers, particularly ovarian, this paper concentrates on the underexplored factors related to macroenvironment and resource which influence cancer outcomes in research predominantly centred around ovarian cancer.

### 2.3. TMR Framework Development

A 3-step process was implemented. Firstly, a conceptual framework was devised using the criteria formulated within the TMR framework and underpinned by the thematic analysis obtained through the narrative review of empirical evidence and rationale. The proposed contribution of each variable within a domain (T domain, M domain and R domain) was based on a hybrid of evidence from prior studies and expert analysis.

Secondly, a Weighted Sum Model (WSM) approach was adopted, where individual factors within each component of TMR were assigned an internal weighting based on perceived relevance within the conceptual framework and to facilitate future feasibility testing and refinement and with a purpose to conduct a prospective validation of the framework. The weights were assigned a priori as provisional values informed by the literature synthesis and expert judgement and were subsequently assessed for acceptability through expert consultation. They were not statistically derived from patient outcomes. Global weights were assigned based on literature synthesis, expert consensus, and the proposed importance of each domain within the conceptual framework, consistent with approaches used in multi-domain healthcare evaluation frameworks in which variables from multiple domains are structured and weighted for comparative assessment. These approaches include the International Prognostic Index (IPI) for non-Hodgkin lymphoma and the Nottingham Prognostic Index (NPI) for breast cancer [14]. Such approaches demonstrate how biological and contextual domains can be considered simultaneously when developing multidimensional frameworks; however, the TMR framework goes beyond traditional prognostic tools by capturing social and system-level influences relevant to survivorship and care experiences.

Thirdly, a multi-criteria decision analysis (MCDA) was developed to integrate and organise multiple dimensions within a single framework. This approach allows the incorporation of variables operating at different levels of influence while maintaining proportional representation of importance within the overall model. As a proof-of-concept exercise, we explored a potential method by which composite scores could be calculated by summing individual factor scores within each domain (T1 to T6, M1 to M7, and R1 to R8), with each factor assigned illustrative values to demonstrate how the framework could be operationalised in future validation studies. A preliminary scoring system was developed in which variables were classified as favourable or unfavourable according to predefined criteria. Binary classification was adopted solely to test whether the proposed domains could be operationalised. Future validation studies should investigate ordinal, continuous and empirically derived representations of individual variables. An illustrative case study was constructed to demonstrate how the conceptual framework may be applied in practice and to assess feasibility of future implementation.

### 2.4. Cross-Sectional Opinion Surveys

Expert review was conducted using two web-based cross-sectional opinion surveys. Survey 1 was designed to evaluate the proposed framework structure, domain composition, and weighting methodology (Supplementary Document S2A). Participants were identified through convenience sampling and included gynaecological oncologists, medical oncologists, patient advocates, and methodological experts from diverse healthcare settings. Contributors were drawn from the Kolkata Gynecological Oncology Trials and Translational Research Group (KolGOTRG), International Gynecologic Cancer Society-affiliated collaborators, the Teesside Ovarian Oncology Research Collaborative (TORCH), and methodological research networks.

The survey consisted of Likert-scale questions assessing agreement with individual framework components and domain weightings. Participants were additionally invited to provide free-text comments regarding framework content, scoring methodology, and potential implementation.

Survey 2 was distributed following revision of the framework based on responses from Survey 1. Participants reviewed the revised framework and a clinical case study illustrating its application. The aim of the second survey was to establish conceptual consensus regarding the framework structure and scoring approach (Supplementary Document S2B).

Survey development, administration, and reporting followed the Consensus-Based Checklist for Reporting of Survey Studies (CROSS) (Supplementary Document S3). Surveys were distributed electronically with reminder invitations to optimise response rates. Consensus was predefined as agreement among at least 70% of respondents. Descriptive statistics were used to summarise responses.

### 2.5. Feasibility Assessment in a Retrospective Pilot Cohort

The final TMR framework was evaluated in a retrospective pilot cohort comprising patients with ovarian cancer treated within a UK regional cancer service. Data were derived from an ongoing local audit undertaken within an area reported to have lower ovarian cancer survival rates compared with national averages.

A standardised data collection form incorporating all TMR variables was developed. Feasibility was assessed by determining the availability and completeness of data required for each tumour, macroenvironmental, and resource domain. Descriptive analyses were undertaken to assess the distribution of domain-specific scores and identify potential barriers to data collection.

## 3. Results

### 3.1. Narrative Review and MCDA Development

Resulting from the narrative review and concept development process, the following tables present a structured conceptual synthesis of the TMR framework, illustrating the multidimensional factors influencing experiences and outcomes across the gynaecological oncology continuum, with a particular focus on ovarian cancer. Table 1 outlines the key tumour-related biological and microenvironmental variables, including stage, histological subtype, homologous recombination deficiency (HRD), and associated molecular characteristics, alongside the conceptual rationale for their inclusion and the evidence underpinning their relevance to ovarian cancer biology.

The table also summarises macroenvironmental determinants, including social, psychological, behavioural, and lifestyle factors that may influence quality of life, healthcare engagement, treatment experiences, and adaptation to disease. In addition, resource-related considerations are presented across diverse healthcare contexts, encompassing healthcare infrastructure, access to specialist services, and policy-level factors that may influence equity of care and patient experiences.

Collectively, these factors provide a structured conceptual framework that integrates biological, social, and systemic domains to support a more comprehensive understanding of ovarian cancer care pathways and survivorship. Table 2 presents the relative weighting structure assigned to each TMR factor using a Weighted Sum Model (WSM), illustrating the proposed relationships among domains within the conceptual framework.

### 3.2. Phase 1 Expert Survey for Consensus on TMR Domain Factors and Weighting

Respondents in the first survey (n = 20/60; 33% response rate) (Appendix A) provided feedback on the framework development process and demonstrated broad consistency with the findings proposed from the narrative review. Suggestions regarding factor inclusion, classification, and weighting were reviewed and incorporated where appropriate. Complete responses (100%) were obtained for all questions.

More than 75% consensus agreement was reached regarding the proposed weighting structure of the TMR framework. Qualitative feedback submitted through open-ended questions was subjected to thematic analysis. Table 2 includes the results of the TMR Global Review Group survey, showing the proportion of participants selecting "agree" or “strongly agree” in relation to the proposed internal weighting of each framework component.

### 3.3. Refinement of the TMR Framework and Illustrative Case Study

Feedback obtained during Phase 1 was thematically reviewed and used to refine the conceptual framework. An illustrative case study was subsequently developed to explore the practical application of the framework within a hypothetical patient context. Following expert feedback, the internal weighting structure was revised (Table 3).

Key modifications included removal of prior lines of therapy from the tumour domain, refinement of the biological factors, and reclassification of performance status from the tumour domain to the macroenvironment domain. Survey feedback indicated that performance status was viewed as reflecting not only clinical functioning but also broader social and healthcare determinants, including access to care and support systems. This recommendation was accepted and incorporated into the revised framework. The domains should not be interpreted as mutually exclusive causal compartments; several variables, particularly performance status, nutritional status and treatment response, represent emergent characteristics arising from interactions between tumour, host and contextual influences.

For illustrative purposes, favourable factors were assigned a score of 1 and less favourable factors a score of 2, with these values multiplied by the corresponding internal weights to demonstrate how different factors may contribute to the overall framework structure. The case study served as a methodological example of how the TMR framework could be operationalised and adapted across contexts. For the purposes of demonstrating operationalisation of the framework, higher illustrative scores represented a greater accumulation of unfavourable TMR characteristics. These values and weighting structures are exploratory and should not be interpreted as validated prognostic thresholds. The framework also highlights the potential variation in the relative influence of tumour, macroenvironmental, and resource-related domains across different healthcare settings. For example, resource-related factors may be less prominent in highly resourced systems with established specialist cancer services, whereas they may play a larger role in contexts with greater constraints in healthcare infrastructure and access.

The case study proposed a best possible TMR score if all is favourable = 30. High risk score was 35–39 and very high ≥ 40. A global TMR score was proposed which accounts for addition of T +M+ R factor scores. M + R score equals 50% of the global weight, and T score the other 50%. Country-/region-specific contexts might change the global weight for T, M and R factors leading to country-specific TMR cut-offs for prognostication. For example, in high-resource settings with accredited centres for OC, R factors are normalised and therefore T and M are the main determinants for OS. In low-resource settings, MR factors may contribute to > 50% global weighting and are key determinants for OS. The best-possible global TMR score if all was favourable = 15.

The following case study was contextualised as an exemplar case; Case 1. 65-year-old, Stage 4, high-grade serous ovarian cancer, HRD with performance status 2 was treated with neoadjuvant chemotherapy followed by Interval Debulking Surgery with favourable CA-125 KELIM score, chemotherapy response score (CRS) 3 and good response to platinum. Patient has body mass index (BMI) = 17, lives in a care home with minimal social support and suffers from anxiety/depression. Socioeconomic score is poor, nearest tertiary cancer treatment centre (optimal resource) is 100 km away resulting in delay in diagnosis and start of treatment and difficulty to access regular follow up.

**Table 3:** Tumour macroenvironment and resource scoring contextualised with a case study.

**Tumour (T) Factor**	**Determinants** **Favourable = 1**	**Determinants** **Unfavourable = 2**	**Factor** **Score**	**Internal** **Weight (IW)**	**Tumour Score** **(IW × FS × 10)**
**T1—Stage**	Stage 1/2	Stage 3/4	2	0.20	4
**T2—Histology**	Serous/endometrioid	Non-serous/LGS	1	0.20	2
**T3—HRD status**	HRD	Non-HRD/HRP	1	0.20	2
**T4—Platinum sensitivity/** CRS */KELIM score	Platinum-sensitive CRS 3	Poor platinum response/resistant CRS 1/2	1	0.20	2
**T5—Disease distribution/PCI**		High burden	2	0.10	2
**T6—Other biology/response** **-Stromal/infiltrative** **-TMB/immune**		Infiltrative/poor biology	1	0.10	1
**Total T score** **T1 + T2 + T3 + T4 + T5 + T6**				**Global weight 0.5**	**13**
**Macroenvironment (M) Factor**	**Favourable = 1**	**Unfavourable = 2**	**Factor** **score**	**Internal** **weight (IW)**	**Macroenvironment score** **(IW × FS × 10)**
**M1—Performance status**	Good/moderate (0–2)	Poor (3–4)	1	0.20	2
**M2—Comorbidity**		Extremes of age, BMI, significant comorbidities,	2	0.20	4
**M3—Socioeconomic status (individual)**		Low socioeconomic status as defined by respective country	2	0.20	4
**M4—Regional/local** **financial position**		Deprived	2	0.10	2
**M5—Social support**		Lack/poor social support system	2	0.10	2
**M6—Culture/health literacy**		Late awareness/late diagnosis from symptom onset	2	0.10	2
**M7—Psychological factors** **(hope/will to survive)**		Poor individual/caregiver will to survival	2	0.10	2
**Total macroenvironment score** **M1 + M2 + M3 + M4 + M5 + M6 + M7**				**Global weight 0.3**	**18**
**Resource (R) factor**	**Favourable = 1**	**Unfavourable = 2**	**Factor** **score**	**Internal** **weight (IW)**	**Resource score** **(IW × FS × 10)**
**R1—**Hospital resources (equipment/beds/radiotherapy HDU/ITU, etc.)		Paucity leading to treatment delay or referral	1	0.20	2
**R2—**Workforce skill density (specialist surgeons or oncologist/100 k population)		Paucity leading to treatment delay or referral	1	0.20	2
**R3—**Access to diagnostics (molecular tests, frozen section, pathology turn-around/quality)		Paucity causing delay in definitive/correct treatment	1	0.20	2
**R4—**Access to targeted therapy or specialised therapy		Paucity causing delay in definitive/correct treatment	1	0.20	2
**R5—**Access to clinical trials		Paucity in access for investigational or newer therapy	1	0.10	1
**R6—**Access to supporting services		Paucity in social care/physio/dietary /palliative care	2	0.10	2
**Total resource score** **R1 + R2 + R3 + R4 + R5 + R6**				**Global weight 0.2**	**11**
**Total TMR score** **(T+ M+ R)**					**42**
**Global TMR score** **[T × 0.5 + (M + R) × 0.5}**				**13** × **0.5 +29** × **0.5** **= 21**	

**Figure 2:**
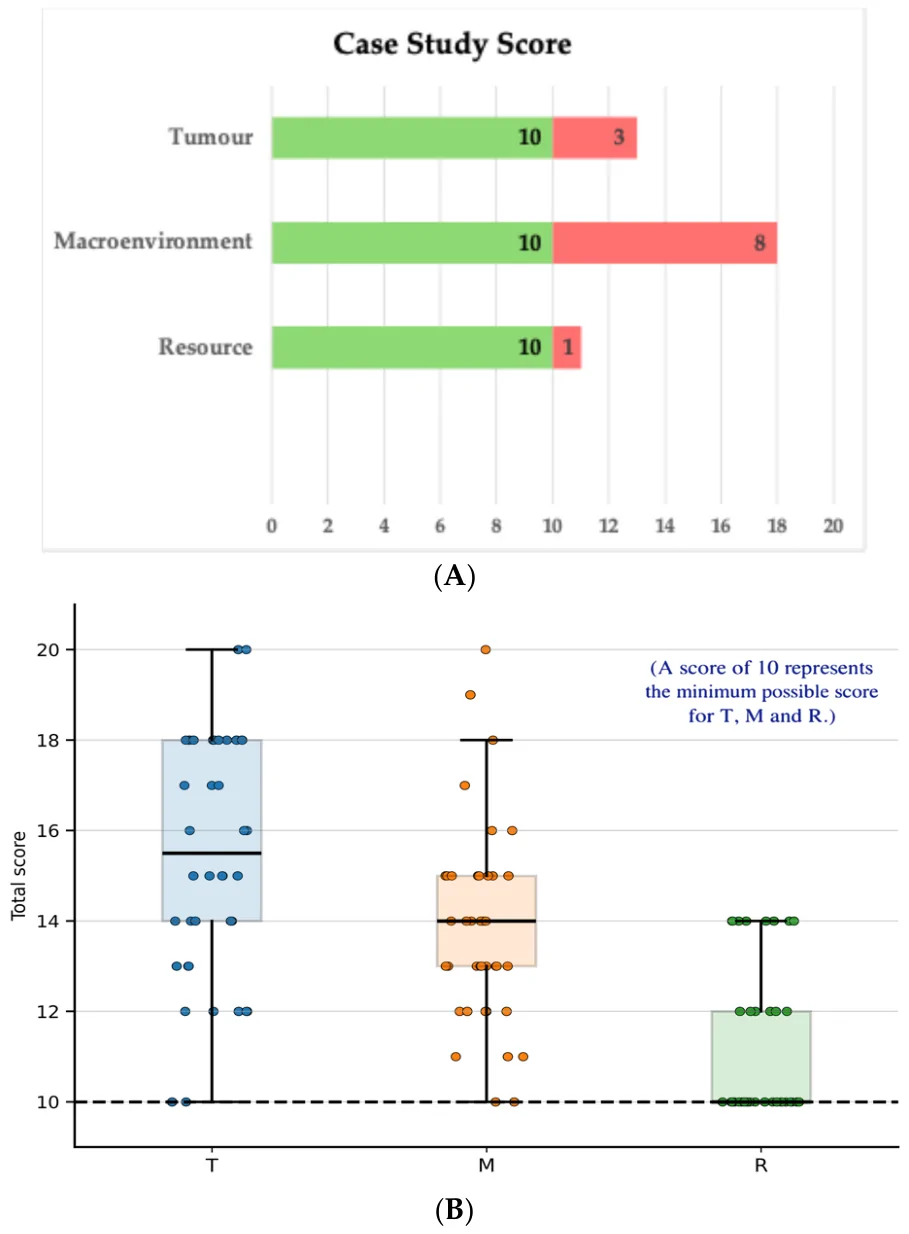
Graphical representation of TMR scores: (**A**) the case study and (**B**) retrospective clinical dataset. (**A**) shows the case 1 domain scores represented in a bar chart with favourable scores displayed in “green” and unfavourable in “red”. Figure created by the authors. In this case, macro-environmental factors were grossly out of the normal range, whereas resource and tumour factors were largely favourable. (**B**) shows the distribution of the T (blue box—Tumour), M (orange box—Macroenvironment) and R (green box—Resource) scores for each of the 42 patients evaluated in the feasibility study dataset. The box and whisker plots demonstrate significant variation in the scores over and above the benchmark of score 10 if all domain variables were favourable. Macroenvironmental scores contributed significantly to the high mean TMR score in this cohort when the resource score was favourable.

Footnote: The unfavourable factors have been highlighted/ticked in red. The total Tumour Macroenvironment and Resource score calculated using the proposed model in this case was 42 and global Tumour Macroenvironment and Resource score was 21. This was based on assigning 50% weighting to tumour factors, 30% weighting to macroenvironmental and 20% weighting to resource factors. We expect that there will be context or country specific alternations to this weighting. The case-specific interpretation was made as follows: high risk for poor overall survival (TMR score > 40); although the tumour score was only 3 points higher (worse) than the best score, the Macroenvironment Resource score was 8 points higher (worse) than best score (Figure 2A). * chemotherapy response score.

### 3.4. Phase 2 Expert Survey for Consensus Development on TMR Table

Phase 2 survey was answered by 44/ 80 participants. Complete response (100%) on all questions was obtained; 94% of participants of the TMR global review group agreed with the revised internal weightings and global TMR scoring model as demonstrated in Table 3 and application to the case study; and 6% of participants were neutral. A total of 100% of participants agreed that prospective validation in a clinical cohort should take place and thematic analysis of the free text comments suggests country-specific adaptations should be carried out when applying the model, particularly within resource factors (Table 4).

### 3.5. Thematic Analysis of Comments from Expert Survey

A thematic analysis was conducted on all comments provided from both phase 1 (13/20 respondents) and phase 2 surveys (21/44 respondents) (Table 4). Comments produced several themes for consideration including country-specific adaptations, tumour-specific adaptations, validation in a clinical cohort and factor-specific comments. This analysis conclusively indicates that validation is required through a retrospective and prospective clinical cohort which will be undertaken in diverse resource settings informing a further research process.

### 3.6. Feasibility of Assigning TMR Score in a Retrospective Cohort

Feasibility Assessment in a Retrospective Pilot Cohort was undertaken. Application of the TMR framework to a retrospective pilot dataset (*n* = 42; September 2021 to May 2022) demonstrated that data could be obtained for all major domains and subdomains in more than 90% of cases, with only a small number of variables showing incomplete availability (Table 5). Tumour Macroenvironment and Resource domain scores are presented in Figure 2B. Within the Tumour domain, unfavourable scores were recorded for two factors in 19.0% of patients, three factors in 21.4%, and four or more factors in 40.5%. Within the Macroenvironment domain, unfavourable scores for two, three, and four or more factors were present in 28.6%, 28.6%, and 23.8% of patients, respectively. Within the Resource domain, nine patients (21.4%) had two or more unfavourable factors.

These findings demonstrate the feasibility of collecting these variables from routinely available clinical data and applying the framework within a real-world ovarian cancer cohort.

## 4. Discussion

Traditionally, anatomical stage alongside histological and molecular classification has provided the primary framework through which cancer outcomes are understood. However, substantial variation exists in patient experiences, survivorship trajectories, and healthcare engagement among individuals with similar tumour characteristics who experience different social and resource contexts. Increasingly, clinical and research guidelines in gynaecological oncology have recognised the importance of incorporating resource-stratified recommendations and principles of equity, diversity, and inclusivity (EDI) into cancer care [53,54,55]. Within this broader context, diversity may be conceptualised as a social biomarker reflecting the interaction between individuals and the structural conditions within which they receive care. The TMR framework was developed to capture these interdependent influences by integrating tumour, macroenvironmental, and resource-related domains into a single conceptual structure. Rather than focusing exclusively on biological disease characteristics, the framework offers a holistic lens through which cancer care and survivorship can be understood across diverse healthcare settings and potentially adapted for different cancer types.

Existing clinical frameworks reviewed during the narrative review phase, including the KELIM model, AGO score, IPI, and NPI, informed the conceptual grounding of the TMR framework. These models provide important approaches for understanding disease characteristics, treatment response, and clinicopathological variation. The KELIM model focuses on tumour chemosensitivity through CA-125 elimination kinetics during chemotherapy in ovarian cancer, while the AGO score supports assessment of factors associated with secondary cytoreductive surgery in recurrent ovarian cancer. Similarly, the IPI incorporates age, stage, and performance status in aggressive lymphoma, and the NPI considers tumour size, nodal involvement, and histological grade in breast cancer. Although these approaches have substantial clinical value, they predominantly centre on biological and clinical variables and generally do not incorporate broader macroenvironmental or resource-related influences. Consequently, they offer limited opportunity to examine how systemic, structural, and healthcare access factors interact with tumour biology to shape patient experiences and care pathways. The TMR framework seeks to address this conceptual gap by bringing these domains together within a unified model.

A phased approach was adopted for framework development, incorporating narrative review, concept generation, and multistage consensus building among experts using ovarian cancer as an exemplar condition. Selection of factors within the tumour (T), macroenvironmental (M), and resource (R) domains was informed by literature review and iterative expert discussion. A narrative review methodology was considered appropriate given the heterogeneity and conceptual complexity of evidence relating to ovarian cancer outcomes, survivorship, and global disparities in care. This approach allowed integration of clinical, psychosocial, societal, and system-level evidence that might not readily be captured through more narrowly defined evidence synthesis methods. Although participation in the first expert survey was lower than anticipated, engagement increased substantially in the second phase, suggesting growing familiarity with and acceptance of the conceptual framework.

The weighting approach incorporated within the framework should be understood primarily as a methodological tool for organising and illustrating the relative prominence of concepts rather than as a quantitative prediction mechanism. Expert consultation provided an opportunity to assess whether the proposed domains and their internal structure reflected contemporary clinical understanding and practical experience. High levels of agreement among experts regarding the relative importance of factors within each domain suggest that the framework possesses conceptual credibility and face validity. Importantly, expert feedback supported the principle that biological, social, and systemic influences should be considered together rather than as isolated determinants of cancer care and survivorship. Future work should explore how the framework can be refined and applied across different contexts while maintaining its conceptual integrity.

Within the tumour domain, stage, HRD/BRCA status, and histological subtype were consistently identified as important biological dimensions relevant to understanding variation in ovarian cancer pathways and treatment experiences [19,20,21]. Other factors, including platinum response, stromal characteristics, and tumour mutational burden, were also recognised as relevant biological considerations, although their relative significance may evolve as evidence develops [23,24]. Residual disease following surgery was not included as a standalone component because it was considered conceptually dependent upon interactions between tumour characteristics and resource-related capacity. Feedback from the expert review process also highlighted the potential value of adapting the framework to specific tumour subtypes, suggesting that future iterations may benefit from disease-specific contextualisation rather than solely organ-based application.

There was unanimous agreement among experts regarding the importance of incorporating macroenvironmental and resource domains into the framework. The macroenvironmental domain encompasses socioeconomic status (SES), quality of life, and wider social determinants of health (SDOHs), including income, education, employment, and social vulnerability. SES reflects a complex intersection of social advantage and disadvantage that influences health experiences through multiple pathways, including deprivation, discrimination, and healthcare accessibility [26,27].

The social epidemiology literature consistently demonstrates that cancer experiences are shaped by wider structural determinants of health. Findings from the WHO Commission on Social Determinants of Health emphasise the relationship between social conditions and health inequalities, highlighting mechanisms such as delayed diagnosis, differential treatment access, and cumulative life-course exposures [28]. Ovarian cancer may be particularly sensitive to these influences because access to specialist assessment and tertiary care services often plays a central role in care delivery. Similarly, broader social epidemiological research demonstrates a graded relationship between social position and disease burden, indicating that inequalities are present across the social spectrum and are not limited to the most deprived populations [30]. Eco-social theory further conceptualises disease as the biological embodiment of social and structural conditions across the life course, recognising factors such as racism, disadvantage, and housing insecurity as important influences on health and healthcare experiences [29].

Financial circumstances constitute an important component of the macroenvironment because they shape an individual’s capacity to engage with healthcare systems and manage the practical consequences of cancer care. The literature on financial toxicity demonstrates that many individuals experience significant direct and indirect costs associated with cancer treatment, including travel, loss of income, hospital attendance, and supportive care requirements [25,31]. Financial burden therefore represents not simply an economic issue but also a broader social experience that can influence wellbeing, healthcare engagement, and survivorship.

Psychosocial factors are another key component of the macroenvironmental domain. Social support, living arrangements, and family or community networks contribute to an individual’s ability to navigate treatment, manage uncertainty, and adapt to illness [36,37]. Although relatively underexplored in ovarian cancer, evidence suggests that strong support structures may facilitate engagement with treatment and supportive care services. The TMR framework therefore recognises social relationships as a potentially important contextual influence on the cancer experience.

Lifestyle-related factors, including nutritional status, diet, body mass index (BMI), physical activity, and dietary quality, are incorporated within the framework as indicators of the broader context in which health behaviours occur [38]. These variables frequently intersect with socioeconomic conditions, access to resources, and opportunities to engage in health-promoting behaviours. Evidence suggests that women with ovarian cancer often experience challenges relating to physical activity and dietary quality, particularly in older age groups [36]. Their inclusion within the framework reflects the interconnected relationship among biological health, social environment, and lived experience.

Performance status, demographic characteristics, and comorbidity were positioned within the macroenvironmental domain following expert review. While commonly treated as clinical variables, these factors are also influenced by wider social and healthcare contexts. Age-related differences in ovarian cancer experiences have been documented, with older women often facing additional challenges associated with comorbidity and healthcare access [56]. Similarly, demographic differences have been observed across racial and socioeconomic groups, reflecting broader patterns of inequality within healthcare systems [40]. The framework therefore recognises these characteristics as existing at the intersection of biological, social, and structural influences.

Culture, health literacy, and language are also incorporated into the macroenvironmental domain because they influence how individuals understand health information, access services, communicate with healthcare professionals, and participate in decision making [39]. Psychological constructs, including hope, coping, and adaptation to illness, further shape how people experience and respond to cancer. Together, these factors illustrate the multidimensional nature of survivorship and reinforce the importance of considering both individual and contextual influences within a unified conceptual framework.

The resource domain captures healthcare system characteristics that may shape access to care, service delivery, and patient experience across diverse settings. Country income levels and health expenditure provide a broad systems perspective, reflecting variations in healthcare infrastructure, workforce capacity, and service availability [43,45]. Global disparities in cancer care remain substantial, with the majority of cancer-related deaths occurring in low- and middle-income countries [32]. The framework therefore recognises resources as a central contextual component rather than simply a background characteristic of healthcare systems.

At the organisational level, factors such as specialist gynaecological oncology centres, workforce expertise, critical care capacity, and surgical infrastructure may influence how care is delivered and experienced. Previous research has highlighted variation in cancer services both internationally and within individual countries, including the United Kingdom [47,48]. The framework does not seek to quantify the impact of these factors but rather to acknowledge their importance within the wider system of care.

Similarly, access to advanced diagnostics and treatment technologies, including specialised imaging, frozen section pathology, and highly specialised surgical procedures, varies considerably between institutions, regions, and countries [51,52]. Resource-related differences may therefore shape opportunities for diagnosis, treatment planning, and care delivery. Other system-level considerations, including pathology turnaround times, service coordination, and availability of supportive and voluntary-sector services, are also included within the framework because they contribute to the broader ecosystem of care. These factors may also interact closely with macroenvironmental influences, particularly social support and quality of life.

Several limitations should be acknowledged. First, the framework is consensus-derived and should be viewed as a conceptual model rather than an empirically established representation of cancer care pathways. Second, whilst the two small cross-sectional opinion surveys were adopted for gaining expert consensus in areas of uncertainty, predictive accuracy and clinical utility are required validation. As closed professional network groups were used to determine the cross-sectional sample of participants, there is a limitation of equity of responses between high- and low-income settings. The weighting structure reflects expert judgement and may be influenced by disciplinary perspectives, institutional contexts, and geographical variation. Third, the Weighted Sum Model and MCDA necessarily simplifies relationships between domains and may not fully capture the dynamic and interacting nature of biological, social, and systemic influences. The methodology can assume linearity with additive independence and proportional contribution of variables to prognostic outcome however ovarian cancer prognosis is biologically complex and may present nonlinear interactions between tumour biology/other factors. A weighted additive model may therefore simplify dynamic interdependencies between prognostic domains. The framework should therefore be viewed as a heuristic device that facilitates conceptual integration rather than a definitive representation of causal relationships. Finally, although the framework includes quality of life and broader contextual determinants, additional work is required to incorporate patient and public perspectives, which may differ from clinically derived priorities.

The authors acknowledge that this work represents an early-stage conceptual and methodological exploration. The purpose of the TMR framework is not to provide a clinically deployable prediction model but to offer an integrative approach through which tumour, macroenvironmental, and resource-related influences can be considered simultaneously. The intention is to stimulate further investigation to refine variable selection, qualitative research involving key stakeholders to re-define Weighted-Sum-Models and derive prognostic stratification in retrospective/prospective datasets from diverse-population/healthcare settings. Understanding the interplay between TMR factors may address several questions on centralisation/decentralisation of care and population-specific cancer policy/prioritisation strategies.

A key limitation of the present framework is that the TMR domains and proposed weightings have not yet been validated using conventional statistical survival methods. Future studies will therefore evaluate tumour, macroenvironmental and resource factors using multivariable proportional/non-proportional hazards regression, allowing their independent contribution to survival to be quantified statistically while adjusting for established clinical and pathological prognostic variables. This will provide hazard ratios and confidence intervals and enable assessment of whether incorporating macroenvironmental and resource factors adds prognostic value beyond conventional tumour characteristics. Until such validation is undertaken, the proposed TMR weightings should be regarded as hypothesis-generating rather than as empirically derived prognostic coefficients. An illustration of a probable model is provided in Appendix C.

## 5. Conclusions

The TMR framework presents a novel conceptual model for understanding ovarian cancer survivorship through the integration of tumour-related, macroenvironmental, and resource-related influences. By explicitly incorporating social, structural, and healthcare system determinants alongside biological characteristics, the framework offers a more holistic approach to understanding the complex factors that shape cancer experiences across diverse settings. In doing so, it addresses an important gap in existing approaches by recognising that survivorship is influenced not only by tumour biology but also by the broader social and healthcare contexts within which individuals receive care. The framework therefore provides a structured means of conceptualising the interrelationships among biological, social, and systemic factors and supports more equitable, person-centred approaches to cancer care and survivorship research.

The TMR framework should be viewed as an initial conceptual and methodological contribution rather than a clinically deployable tool. Future work will focus on refining the framework through wider feasibility studies, larger international consensus exercises, and in-depth stakeholder engagement, including qualitative research with patients, carers, clinicians, and policymakers. Further empirical evaluation across diverse healthcare contexts will be important to explore the applicability, transferability, and contextual relevance of the framework, particularly within resource-stratified settings. Such work may also inform refinement of the relative importance assigned to individual framework components and enhance understanding of the relationships among tumour, macroenvironmental, and resource domains.

Ultimately, the value of the TMR framework lies in its ability to provide a shared conceptual language through which researchers, clinicians, policymakers, and patients can examine the multidimensional influences that shape cancer care and survivorship. By bringing together biological, social, and systemic perspectives within a single integrative framework, TMR offers a foundation for future research, service development, health equity initiatives, and resource-adapted approaches to survivorship care across diverse global settings.

## Figures and Tables

**Table 1:** Proposed domain factors for T, M, R, based on narrative review of the topic.

**Tumour (T) Factor**	**Evidence (Ovarian Cancer Specific)**	**Rationale**
Stage (I–IV)	Stage strongly drives survival.	Traditional predictor of overall survival (OS) [15].
HRD status	HRD positive or negative (+/−).	Strong predictive/theragnostic biomarker in ovarian CA [16,17,18].
Histology (high-grade serous vs clear cell)	High-grade serous resulting in worse outcomes.	Important, but not as strong as stage/HRD [19,20].
Tumour mutational burden (TMB)/actionable mutations	May have prognostic signal in ovarian cancer.	Inclusion as adjunct variable, predictor of immunotherapy benefit [21].
Prior lines of therapy	Each additional relapse reduces OS significantly; resistance accrues.	Impacts prognosis, especially in a recurrent setting [22].
Performance status (PS)	Poor PS associated with worse OS independent of biology.	Smaller consistent effect.
**Macro environment (M) Factor**	**Evidence (Ovarian cancer-specific)**	**Rationale**
Socioeconomic status (SES: income, education, employment)	Lower SES linked to delayed diagnosis, poorer OS.	High impact. [23,24,25,26,27,28]
Financial position	Ovarian cancer patients present later, have lower treatment rates. Income to attend appointments/ transport, parking, access to care.	Moderate-strong effect. Understanding out of pocket impact on cancer patients. [29,30,31]
Social support	Studies show patients with stronger support networks are more likely to complete chemotherapy.	Moderately strong and underexplored. [32,33,34,35]
Nutrition status	Malnutrition at diagnosis resulting in higher mortality risk.	Relevant clinical factor linked to SES. [36]
Demographics and comorbidity	Older age resulting in lower OS even stage-matched; comorbidity higher surgical risk.	Moderately strong and underexplored. Hypothesis includes higher R factor may influence this.
Culture/health literacy	Language and cultural barriers linked to later stage at diagnosis in ovarian CA.	Smaller impact however evidenced. [37,38,39,40].
Psychological (hope, will to survive)	Quality-of-life studies show optimism associated with adherence and OS, but evidence weaker.	Smaller impact. Moderately novel concept. [32]
**Resource (R) factor**	**Evidence (ovarian cancer-specific)**	**Rationale**
Country income/health expenditure	Ovarian survival gap	Largest systems driver [41,42,43,44].
Hospital resources (gynaecological oncology centres, surgical capacity, ICU beds)	Optimal cytoreduction improves OS; requires resource-intense centres/ centralisation; disparity between hospitals in the UK.	High medium or low resource settings impact on OS [45,46,47].
Workforce skill density (gynaecological oncologists per 100 k)	Surgery by specialists increases OS.	High impact [46,48,49].
Technology access (HRD testing, frozen section)	Survival improved when availability of technology—limited in low resource settings.	Medium-high impact [50,51,52]
Clinical trial access	Trial enrolment increases therapy options; minority of patients affected. Stratification factors.	Lower impact.
Pathology turnaround and quality	Delay in histopathology prolongs diagnosis/treatment.	Medium impact
Access to support services	Links to third sector in high resource settings where support signposting, onward referral increasing OS.	Lower impact. Novel concept.

**Table 2:** Detailed internal weightings and feedback from survey 1.

T Factor	Internal Weight	Global Weight	Expert Agreement (Survey Feedback)
T1. Stage (I–IV)	0.25		100%
T2. HRD status	0.20		88.3%
T3. Histology (high-grade serous vs others)	0.20		82.4%
T4. Performance status (PS)	0.15		82.4%
T5. Prior lines of therapy	0.10		82.4%
T6. Tumour microenvironment including stromal factors and TMB	0.10		88.3%
Total	1.00	0.5	
M Factor	Internal weight	Global weight	Expert agreement (survey feedback)
M1. Socioeconomic status (SES)	0.20		100%
M2. Demographics (age sex comorbidity)	0.20		82.4%
M3. Financial position	0.15		82.4%
M4. Social support	0.15		94.1%
M5. Nutrition	0.15		76.5%
M6. Culture/health literacy	0.10		70.6%
M7. Psychological factors (hope/will to survive)	0.05		76.5%
Total	1.00	0.3	
R Factor	Internal weight	Global weight	Expert agreement (survey feedback)
R1. Hospital resources (equipment beds radiotherapy, HDU/ITU, etc.)	0.25		100%
R2. Access to diagnostic and specialised therapy (molecular tests, PARP inhibitor/other targeted agents, frozen section, etc.)	0.20		88.2%
R3. Regional/local financial position	0.20		88.2%
R4. Workforce skill density (specialist surgeons/oncologist per 100 k population)	0.15		94.1%
R5. Clinical trial access	0.10		82.4%
R6. Pathology turnaround and quality	0.05		88.3%
R7. Access to support services	0.05		88.3%
Total	1.00	0.2	

**Table 4:** Thematic stratification of comments from Survey 1 and Survey 2.

**Comment Text from Survey 1**	**Survey Area**	**Key Words**	**Theme**
“Factors may also include factors like KELIM, CRS”	T Factor	KELIM. CRS	Agree
“Clear cell, mucinous tumours have a worse prognosis in advanced stages due to chemoresistance”	T Factor	Chemoresistance	Neutral
“Prior lines of therapy should receive a higher scoring as it is a significant factor in recurrent tumours”	T Factor	Methodology	Disagree
“The weightage may be flexible to be country or region specific”	M Factor	Region/Country	Neutral
“All belong to female sex, hence need not be specified”	M Factor	Quantify	Disagree
“Finances and social support is very important. Patient depends on them for treatment and support during recovery”	M Factor	Finance/support treatment	Agree
“Health literacy and cultural factors should be scored high as even patients with good SES defer the recommended treatment and choose alternative medicines in India, Psychological factor again is a very important issue affecting the willingness to complete treatment and have trust in the process.”	M Factor	Region/country treatment Patient-dependent	Neutral
“Would there not be a direct link between socioeconomic status and financial position. Are there two different metrics being looked at for these two factors?”	M Factor	Metrics, direct link Patient-dependent	Neutral
“Important but may be difficult to measure”	R Factor	Weightage	Agree
“Though all of these are extremely important to survival, i believe based on my experience, that access to diagnostic therapy, surgeons skill and pathology turnaround time should get more weightage”	R Factor	Survival/access Weightage	Neutral /disagree
“Quality of pathology and turnaround time is of extreme significance especially in remote and second tier cities hence it should be placed higher. Access to support services could also get a little higher score because the treatment discontinuation rates depend on that”	R Factor	Region/country treatment/quality	Neutral/ disagree
“Completely Agree”	Methodology	Strongly agree	Agree
“Needs validation as proof of concept”	Methodology	Agree, validation	Agree
“Good”	Methodology	Strongly agree	Agree
“The methodology appears to be robust but individual factors need to be fine-tuned.”	Methodology	Individual factors	Agree
“Agreed”	Methodology	Agree	Agree
“Seems Correct”	Methodology	Agree	Agree
“Agree”	Methodology	Agree	Agree
“Relevant points”	Methodology	Strongly agree	Agree
**Comment/text from Survey 2**	**Survey area**	**Key words**	**Theme**
“Country specific adaptations to the weightage from macroenvironmental and resource factors to be carried out”	M and R Factors	Country-specific	Agree
“T2: LGS is unfavourable for chemotherapy response but not for survival”	T Factor	Chemoresistance	Neutral
“Lack of finances, family issues and social support does derail a patient in LMIC from taking the available complete treatment resulting in poor OS”	M Factor	Patient-dependant	Agree
“Prospective clinical validation will make it useful for application in day to day practice”	Methodology	Validation	Agree
“TMR table very convincing and I am confident it will be transformative”	Methodology	Strongly agree	Agree
“Prospective validation would be crucial”	Methodology	Validation	Agree
“It’s a well-conceived hypothesis/model wherein geographic based adjustment factors in regard to R has been considered in the TMR score, it balances judgement on prognosis and outcome across country boundaries” “Calls for validation”	Methodology	Concept validation	Agree

T = Tumour, M = Macroenvironment, R = Resource.

**Table 5:** Feasibility for data completion for T, M, R domains in a retrospective pilot clinical dataset (*n* = 42).

TMR Sub-Domain	Data Availability %
T1—FIGO stage	100.0
T2—Histology	97.6
T3—HRD status	90.5
T4—Treatment response biology (platinum sensitivity/CRS/KELIM)	90.5
T5—Disease burden (disease distribution. PCI)	92.9
T6—Tumour biology (stroma/mutational burden)	92.9
M1—Patient factors—performance status	99.5
M2—Patient factor—comorbidity	97.6
M3—Socioeconomic/home factors	100.0
M4—Geographic deprivation; regional/local financial position	97.6
M5—Social support/communication factors	96.8
M6—Cultural/health literacy	95.2
M7—Patient factors—psychological factors	
hope/will to survive	97.0
R1—Hospital resources—critical care/treatment delays	100.0
R2—Workforce/service delivery	100.0
R3—Access to diagnostic resources	100.0
R4—Access to targeted therapies	100.0
R5—Access to clinical trials	100.0
R6—Access to supportive services	100.0
**Overall T**	93.9
**Overall M**	97.7
**Overall R**	100.0
**Overall TMR**	95.7
**T score (*n* = 42)**	
Mean (SD)	15.50 (2.63)
Median (IQR) range	15.5(4.0) 10–20
**M score (*n* = 42)**	
Mean (SD)	13.83 (2.15)
Median (IQR) range	14.0 (2.0) 10–20
**R score (*n* = 42)**	
Mean (SD)	11.14 (1.66)
Median (IQR) range	10.0(2.0) 10–14
**Total TMR score (*n* = 42)**	
Mean (SD)	**40.43 (3.97)**
Median (IQR) range	**40.0 (5.5) 33–49**

**Figure A1:**
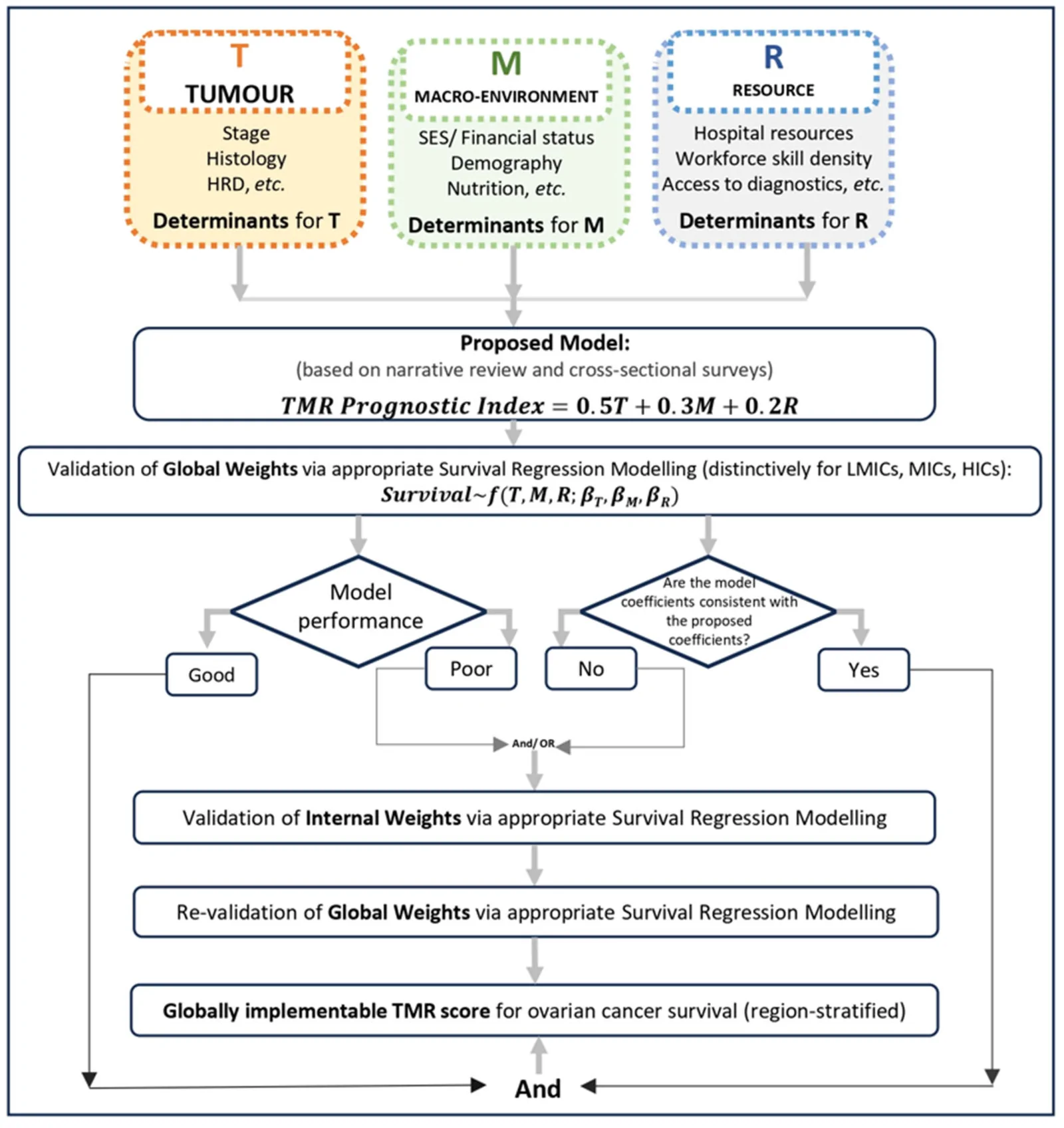
This is a flow diagram illustrating a proposed model for a statistical analysis and validation of the TMR framework. Determinants for Tumour (T) are represented in the orange box, determinants for Macroenvironment (M) are in the green box and determinants for Resource (R) are in the blue box.

## Data Availability

Data available at the following location; 10.6084/m9.figshare.32231448. University Hospitals Tees, Department of Gynecological Oncology and Kolkata Gynaecological Oncology Trials and Translational Research Group.

## Supplementary Materials

The following supporting information can be downloaded at https://www.mdpi.com/article/10.3390/cancers18203299/s1, Document S1 SANRA Checklist, Document S2A Survey 1, Document S2B Survey 2, Document S3 CROSS Checklist.

## Abbreviations

The following abbreviations are used in this manuscript:

SES	Socioeconomic Status
TMR	Tumour Macroenvironment Resource
HRD	Homologous Recombination Deficiency
TMB	Tumour Mutational Burden
TORCH	Teesside Ovarian Oncology Research
QoL	Quality of Life
SDOH	Social Determinants of Health
OS	Overall Survival
BMI	Body Mass Index
HIPEC	Hyperthermic Intraperitoneal Chemotherapy
HDU	High Dependency Unit
ITU	Intensive Care Unit
UK	United Kingdom
MCDA	Multi Criteria Decision Analysis
CRS	Chemotherapy Response Score
KELIM	CA-125 ELIMination rate constant K
PS	Performance Status
BRCA	BReast CAncer gene
WSM	Weighted Sum Model
KolGOTRG	Kolkata Gynecological Oncology Trials and Translational Research Group
IGCS	International Gynaecological Cancer Society

## Appendix A

**Table A1:** TMR global review group (Phase #1 survey).

Name	Organisation	Country	Speciality
Asima Mukhopadhyay	University Hospital Tees/Kolkata Gynecological Oncology Trials and Translational Research Group (KolGOTRG) *	UK/ India	Gyn Oncology
Suday Halder	Medical College and Hospital, Kolkata *	India	Gyn Oncology
Vijay K. Ahuja	Aster International Institute of Oncology, Bengaluru *	India	Gyn Oncology
Ranajit Mandal	Chittaranjan National Cancer Institute, Kolkata *	India	Gyn Oncology
V Annapurna	Sri Shankara Cancer Hospital and Research Centre, Bengaluru *	India	Gyn Oncology
Bijoy Kar	Gujarat Cancer and Research Institute GCRI Ahmedabad *	India	Gyn Oncology
Falak Naz Babbar	University Hospitals Tees, TORCH	UK	Gyn Oncology
Ramesh Shrestha	BPKIHS, Dharan (IGCS network)	Nepal	Gyn Oncology
Nisha Singh	King George’s Medical University Lucknow *	India	Gyn Oncology
Shashank Shekhar	All India Institute of Medical Sciences Jodhpur *	India	Gyn Oncology
Sabuhi Qureshi	Kalyan Singh Super Speciality Cancer Institute, Lucknow *	India	Gyn Oncology
Sarita Kumari	Max Institute of Cancer Care, Max Super specialty Hospital, Dwarka, Delhi *	India	Gyn Oncology
Jitendra Pariyar	Civil Service Hospital of Nepal, Kathmandu (IGCS Network)	Nepal	Gyn Oncology
Diya Sur	Kolkata Gynecological Oncology Trials and Translational Research Group (KolGOTRG) *	India	Patient Advocate/ Survivor
Syamsundar Mandal	Kolkata Gynecological Oncology Trials and Translational Research Group (KolGOTRG) *	India	Epidemiology Biostatistics
Mohamed Mansour Fathi Elsebaei	University Hospitals Tees, Teesside Ovarian Oncology Research (TORCH).	UK	Gyn Oncology
Uddiptya Goswami	SP Medifort Hospital Trivandrum *	India	Medical Oncology
Sushma Agrawal	Sanjay Gandhi Postgraduate Institute of Medical Sciences, Lucknow *	India	Medical Oncology
Nanak Bhagat	Aberdeen Royal Infirmary, Aberdeen	UK	Gyn Oncology
Ally Memon	University of Northumbria	UK	Health Services Management
Lorraine Johnston	University of Northumbria	UK	Public Policy Management

* members of Kolkata Gynecological Oncology Trials and Translational Research Group, India.

## Appendix B

**Table A2:** TMR global review group (Phase #2 survey).

Name	Organisation	Country	Speciality
Asima Mukhopadhyay	Kolkata Gynecological Oncology Trials and Translational research Group (KolGOTRG) *	UK/ India	Gyn Oncology
Diya Sur	KolGOTRG *	India	Patient Advocate
Vijay Kumar Ahuja	Aster International Institute of Oncology, Bengaluru*	India	Gyn Oncology
Syam Sundar Mandal	KolGOTRG*	India	Statistician
Sushma Agrawal	Sanjay Gandhi Postgraduate Institute of Medical Sciences, Lucknow *	India	Clinical Oncology
Annapurna Vadaparty	Sri Shankara Cancer hospital and research centre, Bangalore *	India	
Nisha Singh	King George’s Medical University, Lucknow*	India	Gyn Oncology
Falak Naz Babbar	University Hospitals Tees, TORCH	UK	Gyn Oncology
Bindiya Gupta	UCMS and GTB hospital, Delhi*	India	Gyn Oncology
Jitendra Pariyar	Civil Service Hospital, Kathmandu	Nepal	Gyn Oncology
Sarita Kumari	Max Institute of Cancer Care, Dwarka, Delhi*	India	Gyn Oncology
Daity Bhattacharjee	KolGOTRG*	India	Statistician
Sabuhi Qureshi	Kalyan Singh Super Specialty Cancer Institute, Lucknow*	India	Gyn Oncology
Babita Kataria	National Cancer Institute—Jhajjar, AIIMS Delhi*	India	Medical Oncology
Nanak Bhagat	Aberdeen Royal Infirmary, Aberdeen	UK	Gyn Oncology
Chinmoy K Bose	Netaji Subhas Chandra Bose Cancer Hospital, Kolkata *	India	Gyn Oncology
Shashank Shekhar	All India Institute of Medical Sciences, Jodhpur *	India	Gyn Oncology
Raja Pramanik	All India Institute of Medical Sciences, Delhi *	India	Medical Oncology
Sarita Kumari	Max Institute of Cancer Care, Dwarka, Delhi *	India	Gyn Oncology
Aarthi S Jayraj	Gynaecological Oncology, NCI, Jhajjar *	India	Gyn Oncology
Mubiru Musa	Kawempe National referral hospital, Kampala	Uganda	Gyn Oncology
Aisha Mustapha	Ahmadu Bello University, Zaria	Nigeria	Gyn Oncology
Khalid Abdallah	University Hospitals Tees, TORCH	UK	Gyn Oncology
Uddiptya Goswami	SP Medifort Hospital, Thiruvnanthapuram *	India	Medical Oncology
Mohamed Elsebaei	University Hospitals Tees, TORCH	UK	Gyn Oncology
Ganiy Abdulrahman	University Hospitals Tees, TORCH	UK	Gyn Oncology
Ally Memon	Northumbria University	UK	Health Services Management
Bijoy Kar	Gujrat Cancer Research Institute, Ahmedabad *	India	Gyn Oncology
Suday Halder	Medical College, Kolkata*	India	Gyn Oncology
Vandana Jain	Rajiv Gandhi Cancer Institute and Research Centre, Delhi *	India	Gyn Oncology
Santanu Kumar Tripathi	Jagannath Gupta Institute of Medical Sciences and Hospital, Kolkata/KolGOTRG *	India	Clinical Pharmacology
Sandipan Chowdhuri	Saroj Gupta Cancer Centre and Research Institute Kolkata/ KolGOTRG *	India	Gyn Oncology
Amit Sehrawat	All India Institute of Medical Sciences, Rishikesh	India	Medical Oncology
Ashok Kumar Padhy	Aacharya Harihar PG Institute of Cancer, Cuttack	India	Gyn Oncology
Deepika Dhananjayan	Sri Ramachandra Medical College, Chennai	India	Gyn Oncology
Rema Prabhakaran Nair	Regional Cancer Centre Thirvananthapuram	India	Gyn Oncology
Rakesh Kumar Gupta	All India Institute of Medical Sciences, Raipur	India	Pathology and Lab medicine
Anitha Thomas	Christian Medical College Vellore	India	Gyn Oncology
Biman Kumar Chakrabarty	Saroj Gupta Cancer Centre and Research Institute Kolkata/ KolGOTRG *	India	Gyn Oncology
Manisha Vernekar	Chittaranjan National Cancer Institute Kolkata *	India	Gyn Oncology
Pradip Kumar Saha	PGIMER, Chandigarh	India	Gyn Oncology
Akansha Choudhary	All India Institute of Medical Sciences, Bhopal	India	Medical Oncology
Suryakanta Jayasingh	Aacharya Harihar PG Institute of Cancer, Cuttack *	India	Gyn Oncology
Faraz Vali	HCG Cancer Centre, Vadodara	India	Gyn Oncology

* members of Kolkata Gynecological Oncology Trials and Translational Research Group, India.
