# Supplementary material for: Developing a Conceptual Framework: Integrating Tumour, Macroenvironment and Resource (TMR) as a Composite Determinant of Ovarian Cancer Outcome in Diverse Resource Settings
Source: Cancers (Basel). 2026 Oct 13;18(20):3299. doi: 10.3390/cancers18203299 (PMC13702657; doi:10.3390/cancers18203299)
Supplement: Supplementary file 1 [file cancers-18-03299-s001.zip › Document S2B.pdf]

Consensus on updated TMR (Tumour/Macro-environment/Resource) table

Dear Participants,

We had circulated the first draft of the TMR concept survey in December 2025. Based on the feedback received so far, we have revised the weighting for the TMR scores and modified the table. An example of a case study using the TMR score is attached in this survey.
We invite you to participate in this final short survey (5 minutes) to find out whether you agree with this concept and if you will have any comments.

This table will be published this soon in Cancers and participant names will be acknowledged in a Table. It is likely to be referenced in future works including GCIG and Lancet Commission.

Asima Mukhopadhyay and Adam Ruddock on behalf of the TMR working group

1. To what extent do you agree with the updated internal weightings and content displayed in Table 1

- ◯ Strongly Agree
- ◯ Agree
- ◯ Neutral
- ◯ Disagree
- ◯ Strongly disagree

2. Do you agree with the concept of TMR

- ◯ Agree
- ◯ Disagree
- ◯ Neither Agree nor Disagree

3. Do you agree that a prospective validation should take place?

- ◯ Agree
- ◯ Disagree
- ◯ Neither Agree nor Disagree

4. Any other comments?

5. Please provide your full name *

6. Please provide your institution and speciality *

This content is neither created nor endorsed by Microsoft. The data you submit will be sent to the form owner.

Microsoft Forms
