# Supplementary material for: Developing a Conceptual Framework: Integrating Tumour, Macroenvironment and Resource (TMR) as a Composite Determinant of Ovarian Cancer Outcome in Diverse Resource Settings
Source: Cancers (Basel). 2026 Oct 13;18(20):3299. doi: 10.3390/cancers18203299 (PMC13702657; doi:10.3390/cancers18203299)
Supplement: Supplementary file 1 [file cancers-18-03299-s001.zip › Document S2A.pdf]

A pilot physician survey to develop consensus on a concept of TMR score

A pilot physician survey to develop consensus on a concept of TMR score for determination of survival outcome in ovarian cancer

Globally, there is significant disparity in ovarian cancer survival rates between different countries and even regional differences within a country. We propose to develop a concept of Tumour, Macroenvironment and Resource (TMR) as a composite score, that reflects internal weightings of all components for better prediction of ovarian cancer survival.

While tumour biology, stage and treatment responses are critical, we hypothesize that these factors possibly account for only 50% of the overall survival. The remaining 50% are variably shaped by external influences, divided into macroenvironmental factors including social determinants of health (approximately 30%) and resource-based factors (20%). Individually, each of the T, M and R domains are a composite of several factors which vary in weightage and may be context specific. By integrating these broader determinants into cancer survival and survivorship models, we propose to enhance understanding of cancer care, particularly in ovarian cancer which has one of the highest mortality rates amongst gynaecology cancers globally.

We kindly seek your views on our proposed weighted scores of each factor within the TMR framework.

Tumour (T) Factors

T Factor	Internal Weight
**T1** - Stage (I–IV)	0.25
**T2** - HRD status	0.20
**T3** - Histology (high-grade serous vs clear cell etc)	0.20
**T4** - Performance status (PS)	0.15
**T5** - Prior lines of therapy	0.10
**T6** - Tumour microenvironment including stromal factors and TMB	0.10
**Total**	**1.00**

1

How strongly do you agree or disagree with the internal weightings given to the following tumour factors?
*

	Strongly Agree	Agree	Neutral	Disagree	Strongly Disagree
Stage (I-IV) **0.25**	○	○	○	○	○
HRD status **0.20**	○	○	○	○	○
Histology (high-grade serous vs others) **0.20**	○	○	○	○	○
Performance status (PS) **0.15**	○	○	○	○	○
Prior lines of					

therapy **0.10**	◯	◯	◯	◯	◯
Tumour microenvironment including stromal factors and TMB **0.10**	◯	◯	◯	◯	◯

2

Please provide any comments below

Macro-environment (M) Factors

M Factor	Internal Weight
**M1** - Socio economic status (SES)	0.20
**M2** - Demographics (age sex comorbidity)	0.20
**M3** - Financial position	0.15
**M4** - Social support	0.15
**M5** - Nutrition	0.15
**M6** - Culture/ Health Literacy	0.10
**M7** - Psychological factors (hope/ will to survive)	0.05
**Total**	**1.00**

3

How strongly do you agree or disagree with the internal weightings given to the following factors? *

	Strongly agree	Agree	Neutral	Disagree	Strongly disagree
Socioeconomic status (SES) **0.20**	○	○	○	○	○
Demographics (age sex comorbidity) **0.20**	○	○	○	○	○
Financial position **0.15**	○	○	○	○	○
Social support **0.15**	○	○	○	○	○
Nutrition **0.15**	○	○	○	○	○
[illegible]					

Culture/Health Literacy **0.10**	◯	◯	◯	◯	◯
Psychological factors (hope and will to survive) **0.05**	◯	◯	◯	◯	◯

4

Please provide any comments below

Resource (R) Factors

R Factor	Internal Weight
**R1** - Hospital resources (equipment beds radiotherapy, HDU/ITU etc)	0.25
**R2** - Access to diagnostic and specialised therapy (molecular tests, PARP inhibitor/other targeted agents, frozen section etc)	0.20
**R3** - Regional/local financial position	0.20
**R4** - Workforce skill density (specialist surgeons/oncologist per 100k population)	0.15
**R5** - Clinical trial access	0.10
**R6** - Pathology turnaround and quality	0.05
**R7** - Access to support services	0.05
**Total**	**1.00**

5

How strongly do you agree or disagree with the internal weightings given to the following factors? *

	Strongly Agree	Agree	Neutral	Disagree	Strongly Disagree
Hospital resources (equipment beds radiotherapy, HDU/ITU etc) **0.25**	◯	◯	◯	◯	◯
Access to diagnostic and specialised therapy (molecular tests, PARP inhibitor/other targeted agents, frozen section etc) **0.20**	◯	◯	◯	◯	◯
Regional/local financial position **0.20**	◯	◯	◯	◯	◯
Workforce skill density (specialist surgeons/oncologist per 100k population) **0.15**	◯	◯	◯	◯	◯
Clinical trial access **0.10**	◯	◯	◯	◯	◯
Pathology turnaround and quality **0.05**	◯	◯	◯	◯	◯
Access to support services **0.05**	◯	◯	◯	◯	◯

6

Please provide any comments below

7

Methodology:
Factors will be scored either 1 or 2 and multiplied by the internal weight in each factor.

Scores for each factor will be added together (e.g. T1 + T2 + T3 + T4 + T5 + T6)

Total TMR scores will be multiplied (e.g. T*M*R = Overall Survival (OS) score).

Please provide feedback below on the proposed methodology; *

Your Details:

8

Please provide your full name: *

9

Organisation: *

10

Country: *

11

Speciality: *

12

Do you agree to your name being included in the contributors of this paper: *

◯ Yes

◯ No

This content is neither created nor endorsed by Microsoft. The data you submit will be sent to the form owner.

Microsoft Forms
